# A Rare Case of Pulmonary Artery Sling with the VACTERL Association in a 20-Month-Old Infant

**Published:** 2017-07

**Authors:** Yazdan Ghandi, Akbar Shafiee, Mehrazad Sharifi, Najmeh Sadat Bolandnazar

**Affiliations:** 1 *Amir Kabir Hospital, Arak University of Medical Sciences, Arak, Iran. *; 2 *Tehran Heart Center, Tehran University of Medical Sciences, Tehran, Iran. *; 3 *Amir Almomenin Hospital, Arak University of Medical Sciences, Arak, Iran. *; 4 *Mafi Military Hospital, Shush, Khuzestan, Iran.*

**Keywords:** *Congenital abnormalities*, *Heart defects, congenital*, *Pulmonary artery*, *VACTERL association*

## Abstract

The VACTERL association, co-occurrence of vertebral, anorectal, cardiac, tracheoesophageal, genitourinary, and limb malformations, is a rare congenital anomaly. Several cardiac anomalies have been reported as a part of the VACTERL association, particularly ventricular and atrial septal defects. Pulmonary artery sling is a rare congenital abnormality in which the left pulmonary artery arises from the right pulmonary artery. This anomaly is not frequently observed in the VACTERL association and has been rarely reported. A 20-month-old girl was admitted to our hospital due to pneumonia in the right lung, which had pulmonary artery sling as a part of the VACTERL association. Barium meal X-ray showed pressure effects on the esophagus, and computed tomography angiography revealed pulmonary artery sling. Pneumonia management was done. However, the parents of our patient refused to give consent for the surgical correction of this vascular anomaly. Three months after discharge from the hospital, the patient was visited, at which time the parents again refused surgery and treatment for their daughter despite our recommendations.

## Introduction

The VACTERL association, an acronym for the nonrandom co-occurrence of vertebral anomalies (V), anal atresia (A), cardiovascular defects (C), tracheoesophageal fistulae (TE), renal/radial anomalies (R), and limb defects (L), is observed in almost 16 to 20 cases per 100,000 live births.^1^ The most frequent cardiac defects include ventricular septal defects (VSDs), atrial septal defects, and tetralogy of Fallot, and less common defects are truncus arteriosus and transposition of the great arteries. In this case report, we introduce a girl with pulmonary artery sling as a part of the VACTERL association, which is a rare congenital cardiac anomaly.

## Case Report

**Figure 1:**
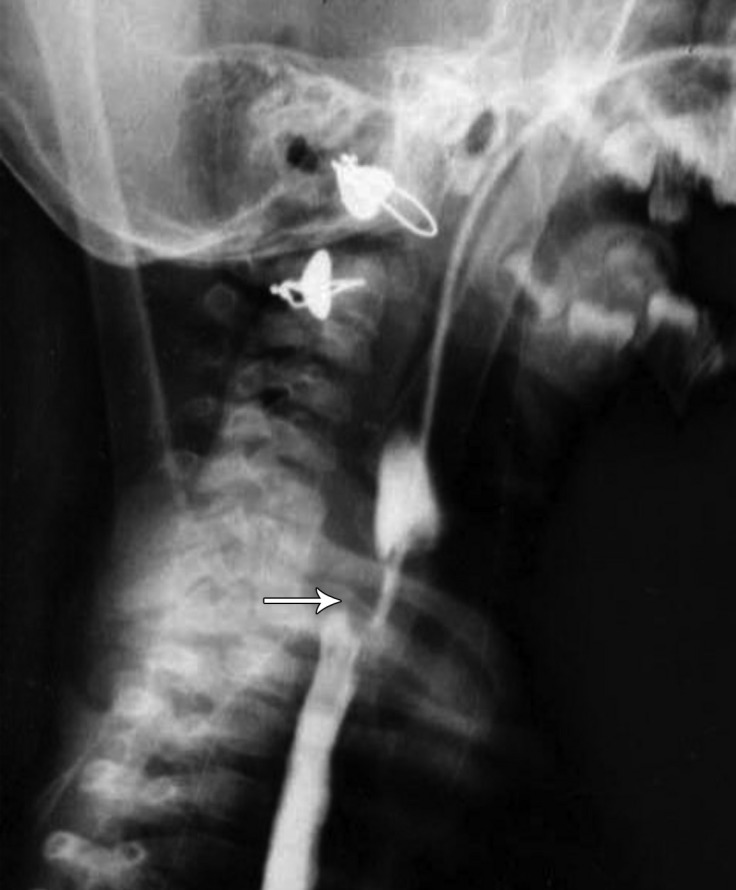
Patient’s barium meal, X-ray, showing pressure effects on the esophagus (arrow).

**Figure 2:**
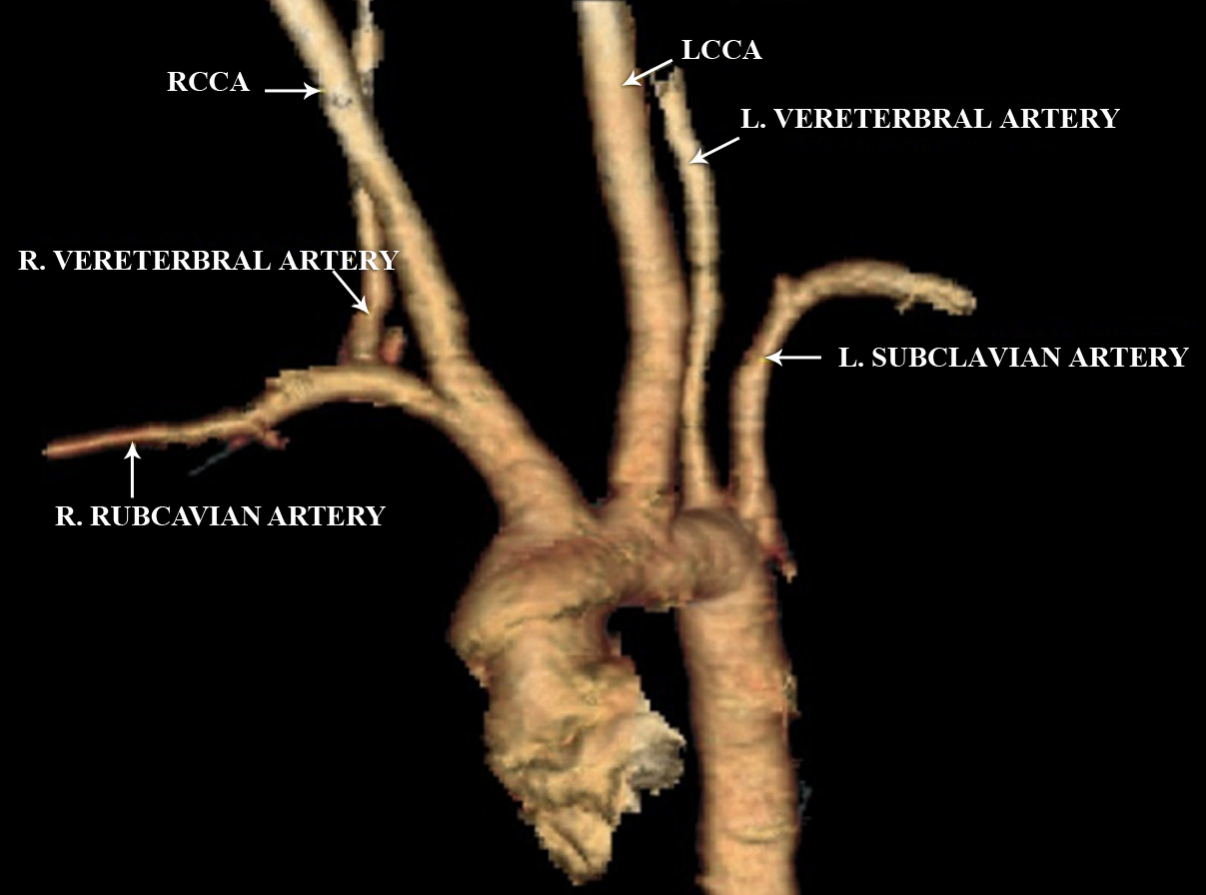
Three-dimensional computed tomography angiography scan of the aortic arch, coronal view, showing the abnormality of the aortic branches and left vertebral artery originating from the aortic arch.

LCCA, Left common carotid artery; RCCA, Right common carotid artery, L, Left; R, Right

**Figure 3:**
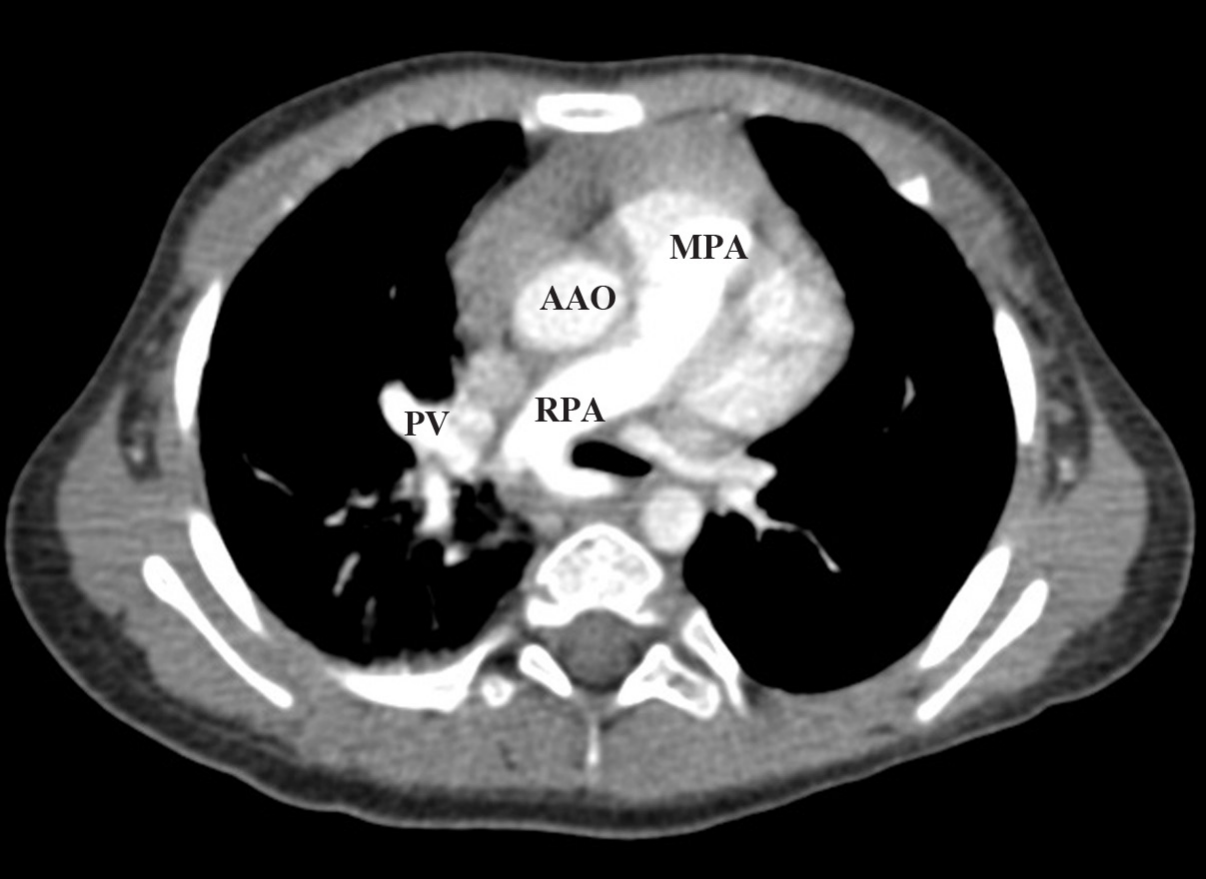
Computed tomography angiography scan of the thorax, showing pulmonary artery sling.

AAO, Ascending aorta; MPA, Main pulmonary artery; PV, Pulmonary vein; RPA, Right pulmonary artery

**Figure 4:**
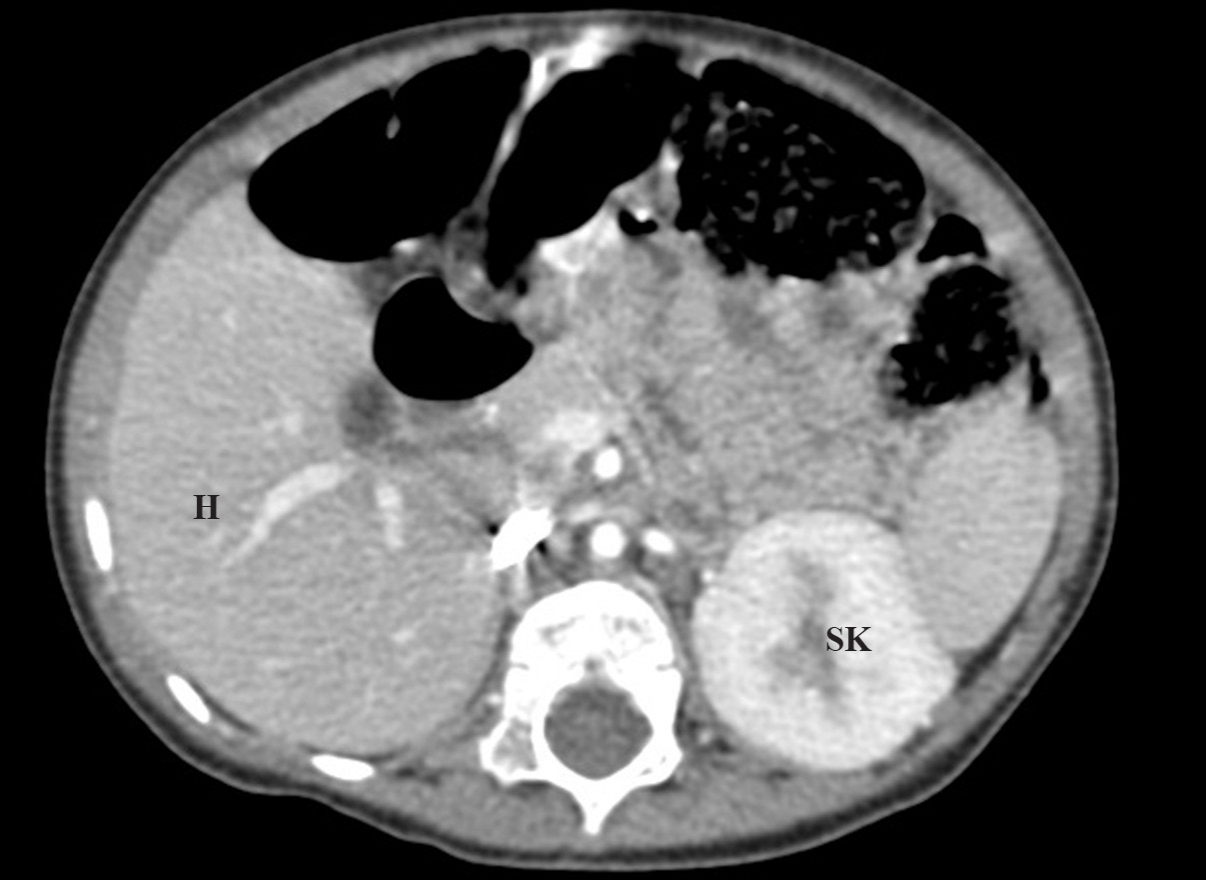
Abdominal computed tomography scan, axial view, showing apparent hepatomegaly and a single kidney.

The patient was a 20-month-old girl, who was admitted due to pneumonia in the right lung. The result of a term normal delivery from a nondiabetic mother, she was the 1st child of the family. The birth weight was 2300 g and the weight at the time of admission was 7500 g. The parents were 3rd-degree relatives, but there was no history of familial diseases or abortion in the family. The patient was initially admitted at the age of 20 days due to imperforated anus with vaginal fistula, which was surgically corrected successfully at the age of 2 months. She was admitted again at 8 months and treated for pneumonia and gastroenteritis. The next admissions were at the age of 11, 14, 16, and 18 months, which were all due to pneumonia in the right lung. She was under treatment for bronchiolitis asthma and gastroesophageal reflux disease (GERD) within this period but had minimal response to these treatments. All the workups for GERD, immunodeficiency conditions, cystic fibrosis, and metabolic diseases were negative. Echocardiography at the age of 2 months revealed a patent foramen ovale (PFO) and a small VSD. Echocardiography at the age of 6 months was reported to be normal. In this admission, the parents revealed that the existence of a single kidney was reported in the gestational ultrasonography and that the patient had suffered 1 episode of urinary tract infection. In physical examination, failure to thrive and rales in the right side were prominent. A barium meal imaging was requested and it demonstrated a pressure effect on the anterior wall of the esophagus (Figure 1). The following echocardiography, performed with suspicion of the VACTERL syndrome, showed no VSD or PFO but a left superior vena cava was observed. Computed scan angiography revealed a pulmonary artery sling and the origination of the left vertebral artery from the aortic arch (Figure 2 and Figure 3). As the patient had a history of imperforated anus, single kidney (Figure 4), and cardiovascular involvement, the VACTERL syndrome was diagnosed for her.

H, Hepatomegaly; SK, Single kidney

## Discussion

Pulmonary artery sling is a rare vascular malformation in which the left pulmonary artery originates from an abnormally placed right pulmonary artery, courses between the esophagus and the trachea, and reaches the left hilum.^2^ Due to the pressure effect of the left pulmonary artery on the trachea, patients can develop respiratory distress, stridor, cyanosis, and dysphagia, as well as obstructive apnea.^3^ Our case is unique inasmuch as the cardiac anomalies seen in the VACTERL association are commonly in the form of septal defects or anomalies of the great arteries. The VACTERL association has been reported to be allied to congenital heart diseases such as VSDs, atrial septal defects, and tetralogy of Fallot. The least common heart defects seen with the VACTERL association are truncus arteriosus and transposition of the great arteries. It is important that cardiac defects be considered an extension of VACTERL. 

 It should be noted that PFOs, VSDs, and left superior vena cava were also present in the present case, but the PFO and the VSD were closed later. Other presentations of the VACTERL association in our patients were anal atresia and unilateral renal agenesis, confirming the diagnosis of the VACTERL association. 

Our patient had several episodes of pneumonia, which may have been due to the presence of the vascular ring and its pressure effects on the right bronchus. The presence of this condition was observed in computed tomography angiography but not in transthoracic echocardiography. Recurrent pneumonia in infants can be a consequence of tracheoesophageal fistulae and chronic aspiration pneumonia. 

Pulmonary artery sling is a rare anomaly of the pulmonary artery that creates pressure on the trachea and the right bronchus, thereby rendering the patient susceptible to pneumonia.^4^ This anomaly is not frequently observed in the VACTERL association and has been rarely reported. The diagnostic methods for the VACTERL association include echocardiography, barium swallow, and computed tomography angiography, and the anomaly can be corrected surgically.^4^^, ^^5^ Unfortunately, the parents of our patient refused to give consent for the surgical correction of this vascular anomaly, despite the recommendations of the treating physicians. 

## Conclusion

It seems that the presence of vascular anomalies is underestimated in patients with the VACTERL association owing to a variety of other complications. Therefore, it should be noted that in patients who present with unusual symptoms of respiratory or upper alimentary tract, evaluation of the vasculature of the thorax may be helpful. 

## Notes:

***This paper should be cited as: ***Ghandi Y, Shafiee A, Sharifi M, Bolandnazar NS. A Rare Case of Pulmonary Artery Sling with the VACTERL Association in 20-Month-Old Infant. J Teh Univ Heart Ctr 2017;12(3):131-133.
